# Psychometric Evaluation of the Pittsburgh Sleep Quality Index in Korean Breast Cancer Survivors: A Confirmatory Factor Analysis

**DOI:** 10.3390/healthcare13192481

**Published:** 2025-09-29

**Authors:** Mi Sook Jung, Moonkyoung Park, Kyeongin Cha, Xirong Cui, Ah Rim Lee, Jeongeun Hwang

**Affiliations:** College of Nursing, Chungnam National University, Daejeon 35015, Republic of Korea; msj713@gmail.com (M.S.J.); lunarnr@cnu.ac.kr (M.P.); huiyongchoe0@gmail.com (X.C.); rimee0901@gmail.com (A.R.L.); jeongeun4813@gmail.com (J.H.)

**Keywords:** cancer survivors, sleep, Pittsburgh Sleep Quality Index, factor analysis, statistical, psychometric validation

## Abstract

**Background/Objectives**: Poor sleep quality is a prevalent and burdensome concern among breast cancer survivors. However, its assessment relies heavily on the Pittsburgh Sleep Quality Index (PSQI), whose latent structure has shown inconsistent support across populations. This study aimed to examine the underlying factor structure and reliability of the PSQI among Korean breast cancer survivors using confirmatory factor analysis. **Methods**: A cross-sectional survey was conducted with 386 non-metastatic breast cancer survivors recruited from a university cancer center in South Korea. Ten competing one-, two-, and three-factor models were identified in previous studies and tested using confirmatory factor analysis with maximum likelihood estimation. Model fit was assessed with χ^2^/*df*, Comparative Fit Index (CFI), Tucker–Lewis Index (TLI), Root Mean Square Error of Approximation (RMSEA), and Standardized Root Mean Square Residual (SRMR), and model parsimony was compared using Akaike Information Criterion (AIC) and Bayesian Information Criterion (BIC). **Results**: The mean global PSQI score was 7.46 (SD = 3.95), and 72.8% of participants were classified as poor sleepers. Among the tested model, a three-factor solution provided the best fit (χ^2^/*df* = 0.795, CFI ≈ 1.000, TLI ≈ 1.000, RMSEA ≈ 0.000, SRMR = 0.017) and achieved the lowest AIC and BIC values. This finding indicates the most favorable balance between fit and parsimony. This three-factor model delineates three distinct but related domains: perceived sleep quality, sleep efficiency, and daily disturbances. The global PSQI demonstrates acceptable reliability. **Conclusions**: These findings support the three-factor structure of the PSQI as the most valid representation of sleep quality among Korean breast cancer survivors. These results underscore the importance of population-specific validation of sleep measures and confirm the clinical utility of this measure as a multidimensional tool for assessing sleep in survivorship care.

## 1. Introduction

### 1.1. Background

Poor sleep quality has become a significant public health issue, with more than 850 million adults affected and experiencing impaired functioning and negative health consequences [1]. This burden is especially severe among cancer survivors, with breast cancer survivors reporting the highest prevalence of insomnia-related symptoms [2]. Evidence from meta-analyses indicates that 43–66% of breast cancer survivors suffer from clinically significant sleep disturbances, exceeding the rates observed in age-matched populations by more than twofold [3,4]. Additional studies have reported that its prevalence peaks during active treatment, affecting nearly 70% of survivors, and remains high at approximately 62% after treatment [4,5]. These elevated rates highlight that disturbed sleep is not a transient effect but a persistent challenge throughout the cancer trajectory.

While advancements in early detection and therapeutic strategies have extended survival among patients with breast cancer, growing attention has been paid to the management of long-term concerns, such as poor sleep [5,6]. Despite its high prevalence and broad impact on fatigue, emotional distress, cognitive inefficiency, and the poor quality of life, sleep problems are underrecognized in oncology practice [3,6,7]. In oncology populations, poor sleep quality is often observed alongside anxiety and depression around the time of diagnosis, and functions as a marker of broader psychological distress, which has implications for survivorship care [2,3,6]. Routine assessments are infrequent and referrals for further evaluation tend to be limited [8]. Moreover, evidence suggests that untreated sleep problems may be linked to recurrence and mortality through disruptions in neuroendocrine and immune functions [6,9].

These challenges necessitate the use of reliable and psychometrically validated tools for evaluating sleep quality. Among the currently available instruments, the Pittsburgh Sleep Quality Index (PSQI) is the most commonly used self-report measure for assessing subjective sleep quality [10]. To capture sleep characteristics, the PSQI aggregates seven components, namely, sleep quality, latency, duration, efficiency, disturbances, medication use, and daytime dysfunction, into a single summed score [11]. Its simplicity and broad applicability have facilitated its use in research and clinical settings [12].

However, the structural coherence of the PSQI has been inconsistently supported in psychometric research. The original developers of the PSQI proposed a single-factor structure based on studies of older adults [11]. However, subsequent research suggested that two- or three-factor models may provide a better representation [13,14,15,16,17,18,19,20,21]. Research on cancer survivors has generally supported a two-factor model [16,17,18,20,21], whereas studies involving older adults and childhood cancer survivors have favored a three-factor model [17,18,19]. These divergent findings suggest that variations in the PSQI structure reflect differences in how individuals perceive and report sleep-related symptoms depending on their demographic, clinical, and cultural backgrounds [22]. This issue is relevant for breast cancer survivors, whose experiences of sleep problems are shaped by a confluence of treatment-related physiological changes, psychological burden, and sociocultural influences [3,6,7]. For example, Otte et al. directly compared the factor structure of the PSQI between Caucasian and African American breast cancer survivors and revealed notable racial variations in the factor structure [23]. These findings suggest that psycho-cultural factors, such as group-specific experiences of poor sleep, distinctive patterns of symptom expression, and culturally embedded interpretations of questionnaire items, may influence how individuals perceive and report their sleep. Such influences can affect the structural validity of sleep assessment tools, such as the PSQI. Building on this premise, this study examined the latent structure of the PSQI among Korean breast cancer survivors by testing the previously proposed factor structures using confirmatory factor analysis (CFA). Identifying the most appropriate structure for this population will enhance the validity and contextual relevance of sleep assessments and support more precise measurements of subjective sleep quality in cancer survivorship research.

### 1.2. Purpose

The purpose of this study was to identify the optimal factor structure of the PSQI among breast cancer survivors by comparing previously proposed one- to three-factor models using CFA based on model fit indices, factor loadings, standardized estimates, and correlations among latent factors.

## 2. Materials and Methods

### 2.1. Study Design

This study used a cross-sectional methodology to explore the factor structure of the PSQI among breast cancer survivors.

### 2.2. Participants

Participants were recruited from a university cancer center located in Daejeon, South Korea. Eligible participants were breast cancer survivors aged 19 years or older who had undergone chemotherapy following surgery and had no prior history of cancer diagnosis or treatment. Additional inclusion criteria included cognitive and language abilities to read and understand the study purpose and provide written informed consent. Individuals were excluded if they self-reported any untreated psychiatric disorders or serious medical conditions that could interfere with their ability to complete the survey. Among 405 breast cancer survivors who expressed interest in the study, 19 were excluded because of ineligibility or incomplete responses. Finally, 386 participants were included in the final analysis.

### 2.3. Measure

Sociodemographic and clinical characteristics of the patients were also assessed. Sociodemographic variables included age, educational attainment, marital status, employment status, health behaviors, and body mass index. Clinical characteristics included the type of surgery, cancer stage, and receipt of adjuvant therapies, such as radiation therapy and hormonal treatment.

Sleep quality was measured using the Korean version of the PSQI [24], a self-report instrument designed to examine perceived sleep quality during the preceding month [11,24]. This instrument comprises 19 items aggregated into seven components: subjective sleep quality, sleep latency, sleep duration, habitual sleep efficiency, sleep disturbances, use of sleep medication, and daytime dysfunction. Each component was rated on a scale of 0–3, following standardized scoring procedures. The sum of these scores yields a global index ranging from 0 to 21 [11]. A global score ≥ 5 indicates poor sleep quality [11]. The PSQI has demonstrated acceptable psychometric properties across diverse populations, including healthy and clinical samples [11,25]. The internal consistency, measured by Cronbach’s alpha coefficients, has been reported to range from 0.70 to 0.83 [25], and was 0.75 in this study.

### 2.4. Study Procedure

A systematic literature search was conducted on 30 July 2025, using the following five databases: Medline, PubMed, CINAHL, Google Scholar, and Web of Science. The search strategy included combinations of “factor analysis,” “CFA,” “EFA,” “factor structure,” “scoring model,” “factor model,” and “PSQI.” The initial search yielded 450 articles. After removing 156 duplicates, we screened the titles and abstracts of the remaining studies and identified nine studies that specifically focused on cancer survivors [13,14,15,16,17,18,19,20,21]. From the nine selected studies, a total of 22 factor structures were identified in the nine selected studies. Upon closer examination, 12 of these models were structurally redundant and exhibited substantial overlap in item composition or factor configuration. Overlapping models were excluded from the analysis to avoid redundancies. Consequently, ten theoretically and empirically distinct models were retained for CFA, including three one-factor models [13,14,15], five two-factor models [13,14,15,16,17,18,19,20,21] and two three-factor models [13,18,19].

### 2.5. Statistical Analyses

Statistical analyses were performed using SPSS (version 29.0; IBM Corp., Armonk, NY, USA) and Mplus 8.7 (Muthén & Muthén, Los Angeles, CA, USA). Data suitability for factor analysis was initially examined through Bartlett’s test of sphericity and the Kaiser-Meyer-Olkin measure of sampling adequacy. Descriptive statistics were used to summarize the demographic and clinical characteristics, and Pearson’s correlation analyses were conducted to assess the relationships among the PSQI component scores.

The final sample size of 386 participants met the conventional criteria and post hoc diagnostics for factor analysis adequacy. A subject-to-variable ratio greater than 10:1 was achieved, which is consistent with general recommendations for factor analysis. Bartlett’s test of sphericity was significant (*p* < 0.001), and the Kaiser-Meyer-Olkin value was 0.764, indicating adequate sampling [26]. All diagonal values in the anti-image correlation matrix exceeded the recommended minimum of 0.50, with the lowest value being 0.69 [27,28]. These results confirmed the suitability of the dataset for factor analysis and supported the retention of all PSQI components.

The factorial structure of the PSQI was examined using CFA. Ten competing CFA models were specified based on prior theoretical frameworks and empirical studies, including three one-factor, five two-factor, and two three-factor structures, all derived from prior theoretical and empirical literature [13,14,15,16,17,18,19,20,21]. CFA was conducted using maximum likelihood estimation with robust standard errors, which accounted for potential non-normality in item distributions. Model fit was assessed using multiple indices: Chi-square statistic and its ratio to degrees of freedom (χ^2^/*df*), Comparative Fit Index (CFI), Tucker–Lewis Index (TLI), Root Mean Square Error of Approximation (RMSEA), and Standardized Root Mean Square Residual (SRMR). According to established recommendations, acceptable model fit was defined as χ^2^/*df* < 2.00, CFI and TLI values ≥ 0.95, RMSEA ≤ 0.06, and SRMR ≤ 0.08 [29].

Model parsimony was examined by comparing the Akaike Information Criterion (AIC) and Bayesian Information Criterion (BIC), with reduced values for these indices indicating an improved fit after accounting for complexity. Standardized factor loadings were inspected to evaluate the item representations of the latent constructs. Factor loadings of ≥0.40 were considered acceptable. Inter-factor correlations were also examined, with values exceeding 0.85 interpreted as evidence of potential conceptual overlap or multicollinearity between the constructs [30].

The internal consistency of the PSQI was examined using corrected item-total correlations and Cronbach’s alpha for the global score and each CFA-identified subscale. A cutoff of 0.70 indicated acceptable reliability [31]. For the two-item subscales, the Spearman–Brown prophecy formula was applied for reliability estimation [32]. These complementary indices ensured a scale-appropriate and methodologically rigorous assessment of internal consistency.

### 2.6. Ethical Consideration

This study was approved by the Institutional Review Board of Chungnam National University (No. 202310-SB-182-01). All participants signed written informed consent forms prior to data collection following a comprehensive explanation of the study’s purpose, procedures, the voluntary nature of participation, and the assurance of confidentiality.

## 3. Results

### 3.1. Sample Characteristics

The sociodemographic and clinical characteristics of the participants are summarized in Table 1. Their mean age was 52.47 years (SD = 7.20) and their average educational attainment was 14.16 years (SD = 6.95). Regarding marital status, most participants were married or living with a partner (n = 315, 81.6%), 8.8% (n = 34) were single, and 9.6% (n = 37) were divorced, separated, or widowed. Regarding employment status, 43.8% (n = 169) were employed, 22.8% (n = 88) were on unpaid leave, 23.1% (n = 89) were retired, and 10.4% (n = 40) were unemployed. Participants reported engaging in moderate-intensity physical activity 2.67 days per week (SD = 2.49) as a health behavior. The mean body mass index was 23.13 (SD = 3.70).

Regarding clinical characteristics, 75.6% (n = 292) had undergone mastectomy and 24.4% (n = 94) had undergone lumpectomy. The cancer stage distribution was as follows: 34.5% of patients were diagnosed at stages 0 and I, 53.4% at stage II, and 12.1% at stage III. Regarding treatment history, 64.0% had received radiotherapy and 32.9% had received hormone therapy.

### 3.2. Descriptive Statistics and Correlation Coefficients for Components of PSQI Scores

Table 2 presents Pearson’s correlation coefficients and descriptive statistics for the global PSQI score and its seven components. The average global PSQI score was 7.46 (SD = 3.95), with sleep latency (M = 1.65, SD = 1.15) and subjective sleep quality (M = 1.47, SD = 0.79) having the highest mean scores among the components. By contrast, the use of sleep medication had the lowest mean score (M = 0.23, SD = 0.72). The use of sleep medication had the lowest mean score, likely reflecting floor effects, as most participants reported little to no use of sleep medication. Overall, 72.8% of participants scored above the suggested cutoff (PSQI ≥ 5), indicating poor sleep quality.

All the components showed significant moderate-to-strong correlations with the global PSQI score, indicating that higher scores reflected poorer overall sleep quality. Intercorrelation coefficients among components ranged from 0.11 to 0.64, representing small-to-moderate associations. No pairwise correlations exceeded 0.85, thereby supporting the discriminant validity of the factor analysis [30].

### 3.3. Confirmatory Factor Analysis

**Figure 1:**
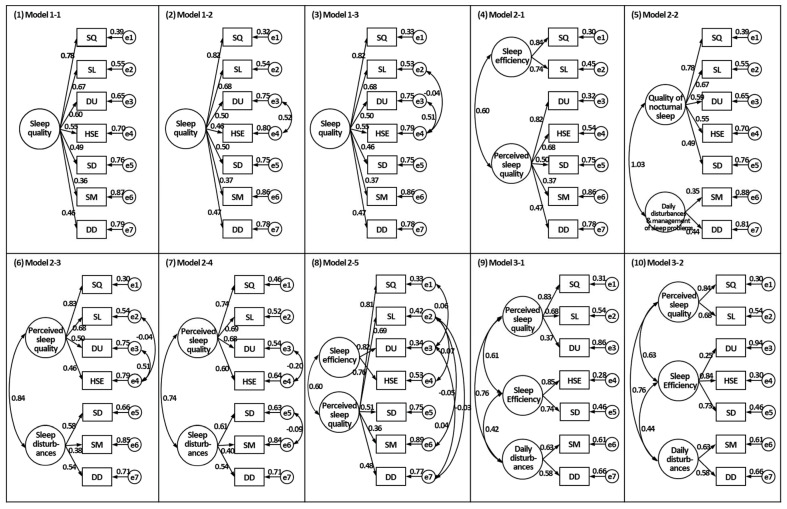
Ten confirmatory factor analysis models of the Pittsburgh Sleep Quality Index. Values represent completely standardized parameter estimates obtained using robust maximum likelihood estimation. Residual variance components reflect the proportion of unexplained variance. Abbreviations: SQ = subjective sleep quality, SL = sleep latency, DU = sleep duration, HSE = habitual sleep efficiency, SD = sleep disturbances, SM = use of sleep medication, DD = daytime dysfunction.

Ten competing CFA models were tested, reflecting a range of theoretically and empirically derived structural configurations of the PSQI (Figure 1). Although all one-factor models (Model 1-1, 1-2, and 1-3) conceptualized global sleep quality as a unidimensional construct [11], they differed in their specifications of correlated residuals [13,14,15]. Among the one-factor models, Model 1-1 demonstrated poor fit (χ^2^/*df* = 5.021, CFI = 0.809, TLI = 0.713, RMSEA = 0.140, SRMR = 0.066). Model 1-2 and 1-3 showed improved fit ((χ^2^/*df* = 1.641, CFI = 0.985, TLI = 0.976, RMSEA = 0.041, SRMR = 0.029 for model 1-2; χ^2^/*df* = 1.699, CFI = 0.985, TLI = 0.974, RMSEA = 0.043, SRMR = 0.030 for model 1-3), but remained inferior to multifactor solutions in global fit indices (Table 3).

Two-factor models generally outperformed one-factor models. Among them, Model 2-3 demonstrated the best overall fit (χ^2^/*df* = 1.370, CFI = 0.993, TLI = 0.986, RMSEA = 0.031, SRMR = 0.025). This finding indicates an excellent model–data correspondence. In contrast, Model 2-1 showed acceptable but relatively weaker fit (χ^2^/*df* = 1.641, CFI = 0.985, TLI = 0.976, RMSEA = 0.041, SRMR = 0.029). This model also exhibited a structurally unbalanced composition, grouping sleep duration and habitual sleep efficiency into a single factor. Despite incorporating several correlated residuals, Model 2-4 showed markedly poor fit (χ^2^/*df* = 11.265, CFI = 0.797, TLI = 0.613, RMSEA = 0.163, SRMR = 0.061). This suggests that the additional complexity failed to resolve the underlying structural misfit. Model 2-2 similarly showed poor fit (χ^2^/*df* = 9.435, CFI = 0.803, TLI = 0.682, RMSEA = 0.148, SRMR = 0.066). Although Model 2-5 exhibited acceptable fit (χ^2^/*df* = 2.298, CFI = 0.981, TLI = 0.951, RMSEA = 0.058, SRMR = 0.028), we excluded it from formal comparisons because its specification relied on modification indices to correlated residuals among indicator loadings on different latent factors, an atheoretical practice that undermines construct validity and increases the risk of capitalization on chance [33].

The three-factor models yielded mixed results. Model 3-1 showed excellent fit across all indices (χ^2^/*df* = 0.795, CFI ≈ 1.000, TLI ≈ 1.000, RMSEA ≈ 0.000, SRMR = 0.017). We report the values exactly as displayed by the software and applied no post hoc model modifications. In contrast, Model 3-2 exhibited moderately weaker fit (χ^2^/*df* = 2.935, CFI = 0.962, TLI = 0.927, RMSEA = 0.071, SRMR = 0.051). Notably, Model 3-1 achieved the lowest AIC and BIC values among all the models tested. This indicates the most favorable balance between fit and parsimony. Although Model 2-3 demonstrated a strong fit and structural simplicity, it fell short of Model 3-1 in statistical performance and conceptual clarity.

A focused comparison of the two best-performing models, Models 2-3 and 3-1, revealed key differences in parameter estimates. In both models, the sleep medication component had standardized factor loadings below the 0.40 threshold, whereas all other components exceeded this value (Figure 1). The inter-factor correlations in Model 2-3 reached 0.84, approaching the conventional upper limit of 0.85. In contrast, those in Model 3-1 were more distinct, ranging from 0.48 to 0.76. These findings indicate that although both models performed adequately, Model 3-1 provided stronger discriminant validity and a more balanced structure.

### 3.4. Reliability

The internal consistency of the PSQI was evaluated using corrected item-total correlations and Cronbach’s alpha. Corrected item-total correlations ranged from 0.30 to 0.65 across the seven components, with all items exceeding the recommended minimum threshold of 0.30. The Cronbach’s alpha coefficients were 0.76 for the overall PSQI, 0.64 for perceived sleep quality, 0.77 for sleep efficiency, and 0.47 for daily disturbances. Although the reliability of the daily disturbances was relatively low, the Spearman–Brown coefficient indicated a modest improvement. Overall, the results suggested acceptable internal consistency for the PSQI in this sample (Table 4).

## 4. Discussion

Sleep disturbance is one of the most prevalent and burdensome symptoms in cancer survivorship and exerts long-term effects on physical, psychological, and functional health. The effective detection of high-risk individuals relies on psychometrically validated instruments that capture the multidimensional complexity of sleep problems. This study examined the latent structure of the PSQI in Korean breast cancer survivors by comparing ten theoretically and empirically derived models. The findings identified a three-factor model as the most parsimonious and best-fitting solution. Its excellent fit likely reflects close alignment with the observed data [29,34]. Although the relative homogeneity of the sample may have contributed, this alone does not explain why the alternative models did not achieve comparable levels of fit [35]. Perfect fit indices can arise when model degrees of freedom are limited [36,37,38]. Importantly, the superiority of the three-factor model was consistently supported across multiple fit indices in a direct comparison with alternative specifications. Overall, these findings affirm the distinct domains of perceived sleep quality, sleep efficiency, and daily disturbances and support the validity of the PSQI as a multidimensional measure for this population.

The three-factor structure identified in this study closely parallels the model proposed by Cole et al. [17]. This configuration differentiates the core aspects of sleep, including subjective perceptions, behavioral patterns, and daytime consequences, thereby providing a more nuanced representation of sleep quality. Unlike prior Western studies that reported excessively high inter-factor correlations (e.g., r = 0.93 between perceived sleep quality and daily disturbances) [13,14,15,18], our findings indicated more distinct constructs, with correlations ranging from 0.48 to 0.76. This pattern supports stronger discriminant validity and may reflect cultural differences in how Korean breast cancer survivors interpret and report sleep-related symptoms. Consistent with this interpretation, a study on Chinese women treated for breast cancer also found an acceptable fit for the three-factor model, although slightly better BIC values were observed for the revised one- and two-factor solutions [13]. Across studies involving Koreans, Chinese, and other racial and ethnic groups, the three-factor structure is broadly relevant, although its stability varies across cultural contexts. Notably, our analyses demonstrated an acceptable model fit without resorting to post hoc modifications, such as residual covariances. This approach preserves theoretical integrity and strengthens both statistical and conceptual robustness [39]. These findings highlight the importance of population-specific validation of latent structures and the necessity for replication studies in diverse cohorts.

Some investigations have recommended adding a residual covariance between sleep duration and habitual sleep efficiency because of their moderate empirical correlation and shared computational basis [17]. Although this adjustment may improve the model fit, it risks obscuring conceptual distinctions. Sleep duration reflects the absolute quantity of sleep, whereas habitual sleep efficiency represents the proportion of time spent asleep [17]. These two components showed moderate correlations in our sample, reinforcing their characterization as related yet distinct constructs. In line with the psychometric guidelines [13], we retained their separation to preserve conceptual clarity. Future research employing longitudinal and cross-cultural designs is required to determine whether this distinction remains stable across populations and time.

The reliability observed in this study (α = 0.76) was consistent with prior reports in the cancer population (α = 0.74–0.83) [7,10,17]. This result supports the PSQI as a stable measure of sleep quality in this context. Among the subscales, sleep efficiency exhibited the highest reliability. This finding indicates that it is particularly well-defined and internally coherent in Korean breast cancer survivors [31,40]. In contrast, daily disturbances exhibited lower reliability, which was reflected in the Cronbach’s alpha and Spearman–Brown coefficients. Although these values were within the range reported in prior studies (α = 0.28–0.83), the reduced internal consistency may reflect contextual or linguistic factors specific to this population [14,25,41,42]. In collectivistic societies such as Korea, individuals may under-report distress or place greater emphasis on daytime functioning over subjective discomfort; this tendency could contribute to the observed pattern [43]. From a clinical perspective, the global PSQI score should be prioritized as the primary indicator for screening and monitoring, and subscales with limited reliability, such as daytime dysfunction, should be interpreted with caution. Future research should refine item wording and structures to strengthen the reliability and cross-cultural relevance of this domain. Beyond the psychometric structure, our findings confirmed the substantial burden of poor sleep among breast cancer survivors. The mean PSQI global score in this sample was 7.46, which exceeded the values reported in a meta-analysis of cancer survivors that examined breast cancer patients before (6.88), during (7.38), and after treatment (6.75) [44]. Moreover, 72.8% of participants were classified as poor sleepers. This prevalence is higher than the 40–60% typically observed in other cancer populations and is consistent with the wide range of 20–76% documented in prior studies of individuals treated for breast cancer assessed using the PSQI [45,46,47,48]. Together, these findings suggest the disproportionate vulnerability of women with breast cancer to sleep problems, and establish poor sleep as a persistent and defining concern in survivorship care.

This study had several notable strengths. To the best of our knowledge, this is the first comprehensive CFA of the PSQI in Asian breast cancer survivors that directly compared both empirically and theoretically derived models. The relatively large sample size and use of robust maximum likelihood estimation improved model performance, whereas the inclusion of multiple fit indices allowed for a rigorous evaluation of model performance. Furthermore, the clinical homogeneity of the sample helped minimize variance attributable to extraneous factors.

This study had some important limitations. First, the cross-sectional design of this study limits the ability to evaluate the stability of the factor structure over time. Second, the daily disturbances subscale showed low internal consistency, which may reduce the accuracy of this domain. Future work is required to refine this subscale and evaluate its reliability in other cultural settings. Third, the reliance on participants from a single institution potentially constrains the generalizability of the findings. Finally, sleep was assessed only with PSQI, a validated self-report instrument. Although essential for capturing perceived sleep quality, prior studies have shown variable correspondence between self-reports and objective measures such as actigraphy and poly-somnography. Future research should incorporate multi-method approaches to provide a more comprehensive evaluation of sleep. Despite these limitations, this study provides a valuable psychometric evaluation of the PSQI in a population where an accurate assessment of sleep quality is critical for improving survivorship care.

## 5. Conclusions

This study provides compelling psychometric evidence for the three-factor structure of the PSQI in Korean breast cancer survivors. Instead of reflecting a single differentiated construct, the instrument captured three related yet distinct domains: perceived sleep quality, sleep efficiency, and daily disturbances. Although the overall reliability was acceptable, the daily disturbances factor demonstrated weaker internal consistency. This indicates the need for refinement to improve the PSQI’s cultural relevance in this population. Beyond these considerations, the high prevalence of poor sleep underscores its role as a persistent and disproportionate challenge for breast cancer survivors. Collectively, these findings affirm that the PSQI is a reliable and clinically informative tool for this population and emphasize the importance of culturally sensitive approaches to sleep assessment in survivorship care.

## Figures and Tables

**Table 1:** General characteristics of participants (N = 386).

Variables	Categories	M (SD) or n (%)
Age, year		51.72 (7.1)
Education, year		14.16 (7.0)
Marital status	Single	34 (8.8)
	Currently married or living with partner	315 (81.6)
	Divorced/separated/widowed	37 (9.6)
Employment state	Employed	169 (43.8)
	Unpaid leave	88 (22.8)
	Retired	89 (23.1)
	Unemployed	40 (10.4)
Physical activities, day		2.67 (2.5)
Body mass index		23.21 (3.7)
Type of surgery	Lumpectomy	94 (24.4)
	Mastectomy	292 (75.6)
Cancer stage	0 & I	133 (34.5)
	II	206 (53.4)
	III	47 (12.2)
Radiotherapy	Yes	247 (64.0)
	No	139 (36.0)
Hormone therapy	Yes	127 (32.9)
	No	259 (67.1)

**Table 2:** Correlations and descriptive statistics of PSQI components (N = 386).

Components	1	2	3	4	5	6	7
1. Subjective sleep quality	-						
2. Sleep latency	0.56 **	-					
3. Sleep duration	0.42 **	0.36 **	-				
4. Habitual sleep efficiency	0.38 **	0.29 **	0.64 **	-			
5. Sleep disturbance	0.41 **	0.30 **	0.19 **	0.25 **	-		
6. Use of sleep medication	0.31 **	0.28 **	0.20 **	0.11 *	0.18 **	-	
7. Daytime dysfunction	0.36 **	0.31 **	0.20 **	0.20 **	0.37 **	0.16 **	-
M (SD)	1.47 (0.79)	1.65 (1.15)	1.34 (1.01)	0.74 (1.05)	1.19 (0.50)	0.23 (0.72)	0.90 (0.91)

* *p* < 0.05, ** *p* < 0.01.

**Table 3:** Goodness-of-fit indices for the factor models of PSQI.

Model	χ^2^/*Df*	*p*	CFI	TLI	RMSEA	SRMR	AIC	BIC
1-1	5.021	<0.001	0.809	0.713	0.140	0.066	6278	6361
1-2	1.641	0.067	0.985	0.976	0.041	0.029	6175	6262
1-3	1.699	0.060	0.985	0.974	0.043	0.030	6176	6267
2-1	1.641	0.067	0.985	0.976	0.041	0.029	6175	6262
2-2	9.435	<0.001	0.803	0.682	0.148	0.066	6280	6367
2-3	1.370	0.179	0.993	0.986	0.031	0.025	6172	6267
2-4	11.265	<0.001	0.797	0.613	0.163	0.061	6269	6363
2-5	2.298	0.019	0.981	0.951	0.058	0.028	6183	6289
3-1	0.795	0.646	1.000	1.000	0.000	0.017	6165	6260
3-2	2.935	0.001	0.962	0.927	0.071	0.051	6191	6286

Abbreviations: χ^2^/*df* = Chi-square statistic and its ratio to degrees of freedom, CFI = Comparative Fit Index, TLI = Tucker–Lewis Index, RMSEA = Root Mean Square Error of Approximation, SRMR = Standardized Root Mean Square Residual, AIC = Akaike Information Criterion, BIC = Bayesian Information Criterion.

**Table 4:** Corrected item-total correlations and internal consistency reliability of the PSQI.

Factor	Components	Corrected Item-Total Correlation	Cronbach’s Alpha	SB
Perceived Sleep Quality	SQ	0.65	0.64	-
	SL	0.54		
	SM	0.30		
Sleep Efficiency	DU	0.55	0.77	0.77
	HSE	0.49		
Daily Disturbances	SD	0.42	0.47	0.54
	DD	0.38		

Abbreviations. SB = Spearman–Brown (prophecy) correction, SQ = subjective sleep quality, SL = sleep latency, DU = sleep duration, HSE = habitual sleep efficiency, SD = sleep disturbances, SM = use of sleep medication, DD = daytime dysfunction.

## Data Availability

The data presented in this study are available upon request from the corresponding author. Owing to participant privacy and ethical restrictions related to the sensitive nature of patient data in our study, the raw data cannot be made publicly available. However, we are willing to provide the minimal dataset necessary for validation upon reasonable request, subject to compliance with ethical and privacy regulations.
